# A blind deconvolution approach to high-resolution mapping of transcription factor binding sites from ChIP-seq data

**DOI:** 10.1186/gb-2009-10-12-r142

**Published:** 2009-12-22

**Authors:** Desmond S Lun, Ashley Sherrid, Brian Weiner, David R Sherman, James E Galagan

**Affiliations:** 1 Phenomics and Bioinformatics Research Centre, School of Mathematics and Statistics, and Australian Centre for Plant Functional Genomics, University of South Australia, Mawson Lakes Boulevard, Mawson Lakes, SA 5095, Australia; 2 Seattle Biomedical Research Institute, 307 Westlake Avenue North, Suite 500, Seattle, WA 98109, USA; 3 Molecular and Cellular Biology Graduate Program, University of Washington, Seattle, WA 98195, USA; 4 Broad Institute of MIT and Harvard, 7 Cambridge Center, Cambridge, MA 02142, USA; 5 Department of Global Health, University of Washington, Seattle, WA 98195, USA; 6 Department of Biomedical Engineering and Department of Microbiology, Boston University, 44 Cummington Street, Boston, MA 02215, USA

## Abstract

We present CSDeconv, a computational method that determines locations of transcription factor binding from ChIP-seq data. CSDeconv differs from prior methods in that it uses a blind deconvolution approach that allows closely-spaced binding sites to be called accurately. We apply CSDeconv to novel ChIP-seq data for DosR binding in *Mycobacterium tuberculosis *and to existing data for GABP in humans and show that it can discriminate binding sites separated by as few as 40 bp.

## Abstract 2

CSdeconv is a novel method for determining the location of transcription factor binding from ChIP-seq data that discriminates closely-spaced sites.

## Background

With the rapidly decreasing cost of DNA sequencing, chromatin immunoprecipitation (ChIP) followed by sequencing of the resulting DNA fragments (ChIP-seq) is fast becoming the most attractive method for the study of genome-wide protein-DNA interaction, yielding advantages such as lower cost, higher resolution, and a lower requirement for input material over the principal alternative, ChIP-chip, which involves hybridization of the immunoprecipitated fragments to a genomic microarray [1-3]. But to harness fully the potential of ChIP-seq, analysis techniques that accurately translate sequencing reads into reliable calls of the genomic locations of the sites of protein-DNA interaction are necessary. To date, a number of such analysis techniques have been developed [2,4-14]. These methods, however, generally do not identify distinct binding sites lying close together (separated by a distance on the order of 100 bp or less), instead interpreting such cases as a single, incorrectly located binding site. Such cases of closely spaced binding sites arise regularly, especially in prokaryotic genomes (see, for example, [15,16]), and an analysis technique capable of making the correct calls is necessary for the full potential of ChIP-seq to be realized.

We present CSDeconv, a computational method that accurately identifies binding sites, including closely spaced binding sites, from ChIP-seq data. In contrast to prior methods that identify binding sites by searching for enrichment peaks in sequenced reads, we recognize that peaks cannot be clearly and distinctly resolved when binding sites are separated by short distances, and we therefore instead use a blind deconvolution approach in which we simultaneously estimate the shape of an enrichment peak as well as the location and magnitude of binding sites. Our work builds on many of the innovations introduced by Valouev and colleagues [4] to the analysis of ChIP-seq data in their method QuEST, including using kernel density estimation [17,18] to estimate the probability density function associated with the location of sequencing reads.

To demonstrate the capabilities of CSDeconv, we have applied it to novel ChIP-seq data for the DosR (dormancy survival regulator) transcription factor in *Mycobacterium tuberculosis *(MTB) and to existing data collected by Valouev and colleagues [4] for the GABP (growth-associated binding protein) transcription factor in humans. The DosR dataset is well-suited to CSDeconv because, in comparison to most mammalian transcription factors, DosR binds only to a small number of sites, allowing the sites to be studied in detail. Moreover, the computational requirements of CSDeconv restrict the number of binding sites that can be analyzed to this scale. Nevertheless, CSDeconv can be applied to mammalian data, and we demonstrate this by analyzing GABP binding over a 2-Mbp segment of human chromosome 19.

In our analysis of DosR binding, we found 24 distinct binding sites distributed over 18 regions, of which 15 regions are upstream of genes whose hypoxic induction has been previously shown to be dependent on DosR [16]. Moreover, our predictions appear spatially accurate with 23 of the 24 predicted sites located within 50 bp of a motif closely resembling that previously identified by Park and co-workers [16]. Notably, four binding sites occur in two closely spaced pairs, and three occur in a closely spaced triplet, and it is clear that these sites cannot be distinguished by using prior peak-calling algorithms. One of the closely spaced pairs occurs in the promoter region of the gene *acr *(Rv2031c), where the centers of the two distinct sites are separated by only 57 bp. That binding occurs at both of these sites was previously established by mobility shift assays [16], and the relative contributions of the two sites to the induction of *acr *by DosR under hypoxia corresponds qualitatively to the relative binding magnitudes established by our algorithm. In our analysis of GABP binding on chromosome 19, we found 23 distinct binding sites distributed over 15 regions. Of the 23 binding sites, 18 are located within 50 bp of a motif resembling that previously identified [4,19].

Owing to the ability of CSDeconv to call closely spaced binding sites, it is capable of achieving a greater level of accuracy, as determined by motif analysis, than do alternative methods when calling the same number of binding sites. We demonstrate this capability by comparing the performance of CSDeconv with MACS [7] and SISSRs [9], two publicly available ChIP-seq peak finding methods.

## Materials and methods

### Density estimation of enriched regions

We divided the genome into *N *nonoverlapping bins. The number of bins *N *was chosen so that the expected number of reads in each bin, assuming a uniform distribution, would be at least 10. For simplicity, we rounded bin sizes up to the nearest 100. For the MTB genome, this resulted in 4,412 nonoverlapping bins, each of length 100 bp, and, for the 2-Mbp segment of human chromosome 19 that we studied, this resulted in 182 nonoverlapping bins, each of length 1,100 bp.

We took reads from a ChIP library and reads from a control library and placed them into these bins. We then calculated the log-likelihood ratio (LLR) for independence of the ChIP distribution from the control distribution for each bin, which is given by

where *n*_ChIP _and *n*_ctrl _are the number of ChIP and control reads in the bin, respectively, and *N*_ChIP _and *N*_ctrl _are the total number of ChIP and control reads in the entire dataset, respectively.

We selected those bins with more ChIP reads than control reads whose LLRs exceeded a certain threshold. For each selected bin, we added 300 bp on either side to ensure that the entire enrichment peak is captured, and we call such a genomic region an *enriched region*. Adjacent or overlapping enriched regions are combined into a single enriched region. Let *k *be the number of enriched regions.

For each enriched region, we applied kernel density estimation with a gaussian kernel. By following the method of [4], we chose kernel bandwidths empirically to be those that yielded good performance. We chose a bandwidth of 30 for IP reads and a bandwidth of 300 for control reads. For enriched region *i*, we obtain four density functions, , , , , for the forward and reverse ChIP reads and the forward and reverse control reads, respectively. We then compute forward and reverse enrichment profiles, sampled at integer position values *m*, according to

and

### Initial peak shape estimation

We make an initial estimate of the shape of an enrichment profile as follows. We aim to select a pulse that is strong (of large amplitude) and narrow, such as to select a pulse that is observed with low noise and that is likely to arise from a single binding site. Thus, for each enriched region, we compute the full width at half maximum (FWHM) and amplitude of the forward and reverse enrichment profiles and compute the average FWHM and amplitude for the region by taking the mean of the forward and reverse values.

We then take the top quartile of the enriched regions according to average amplitude and select the enriched region i* with the smallest average FWHM in this set. In effect, this selects the narrowest peak from among the strongest pulses serving as a good initial estimate of a single binding site. The enriched region i* thus selected is used to compute the initial peak shape.

Specifically, we set the discrete function *h*_0 _and the scalar *m*_0 _according to

where *M*_i* _is the length of the selected enriched region i*. The function *h*_0 _is normalized to a maximal amplitude of unity, and the normalized function describes the initial peak shape.

### Iterative blind deconvolution

We initialize the procedure by setting *h *:= *h*_0_. Then, for each enriched region *i*, we solve

where *M*_*i *_is the length of enriched region *i*, and *α *is a regularization factor that biases solutions with fewer components. The estimates a* and m* are of the amplitudes and positions of the binding sites, and the estimate N* is of the number of the components in the enriched regions.

We solve the minimization problem for each *i *by starting with *N*_*i *_= 1 and solving the minimization over *a*_*i *_and *m*_*i *_by random-restart gradient descent (see, for example, [33]). We then increment *N*_*i *_and solve over *a*_*i *_and *m*_*i *_again, and we continue in this fashion until the objective increases.

For a given (a*, m*, N*), we reestimate *h *by assuming that (a*, m*, N*) are true and estimating the most likely *h; *that is, we solve

which can be solved as a constrained linear least-squares problem, and set *h *:= h*. We repeat this iterative procedure until convergence in *h*.

### DosR ChIP-seq library construction and sequencing

MTB strains H37Rv and H37Rv:Δ*dosR *were grown to early log phase and then exposed to 0.2% O_2 _for 2 hours, as described in [22]. The bacilli were fixed by addition of formaldehyde, lysed with bead beating (6 × 15 seconds with cooling on ice between beats), and DNA sheared by sonication. The extract was incubated with anti-DosR antibodies and run over MagnaBind Protein A coated beads (Thermo Fisher Scientific Inc., Rockford, IL, USA). The antibody-bound complex was eluted from the beads, crosslinking was reversed by the addition of SDS and incubation at 65°C, and DNA fragments were purified by using a QIAquick PCR Purification Kit (QIAGEN Inc., Valencia, CA, USA). The DNA was blunted, and adapters were ligated to each end to facilitate Solexa sequencing. PCR was then used specifically to enrich for DNA fragments with adapter molecules ligated to both ends. DNA obtained from H37Rv was used for the ChIP library, whereas that from H37Rv:Δ*dosR *was used for the control library. Sequencing was carried out by using the Illumina/Solexa Genome Analyzer system, according to the manufacturer's specifications. We obtained a total of 8,361,463 reads in the ChIP library, of which 5,748,148 (68.7%) were aligned (some reads were not aligned, as they were not considered uniquely alignable), and a total of 9,627,826 reads in the control library, of which 6,041,158 (62.7%) were aligned. Reads were aligned as described in [3].

### GABP ChIP-seq dataset

ChIP-seq data for the GABP transcription factor in humans was obtained from Valouev and associates [4]. This dataset contains 7,862,231 aligned ChIP reads and 17,404,922 aligned control reads. We omitted from the dataset all reads that did not lie on chromosome 19 between positions 60,000,000 and 62,000,000, which resulted in 27,800 aligned ChIP reads and 19,930 aligned control reads.

### Software implementation

CSDeconv is implemented by using MATLAB R2009a (The Mathworks, Inc., Natick, MA, USA) and is freely available for nonprofit use [34].

## Results and Discussion

**Figure 1:**
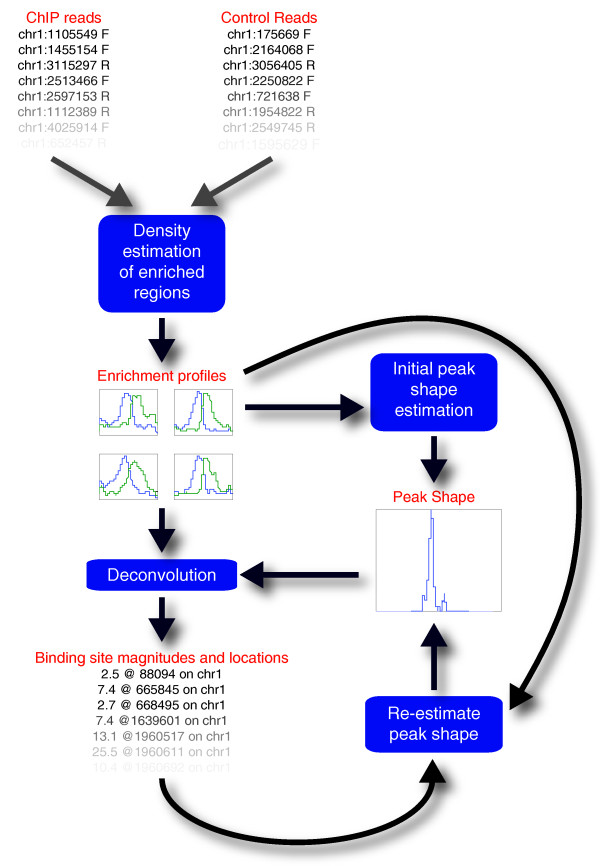
**Overview of CSDeconv**. After an initial stage in which enriched regions are identified and probability density functions associated with ChIP and control read locations are derived, we obtain enrichment profiles that describe the enrichment level throughout each enriched site for both forward and reverse reads. From the enrichment profiles, an initial estimate is made of the shape of an enrichment peak. The peak shape is used to deconvolve the enrichment profiles, deriving binding-site locations and magnitudes, which are then used to reestimate the peak shape, and this iterative cycle is repeated until convergence.

An overview of CSDeconv is shown in Figure 1. CSDeconv begins with an initial stage in which enriched regions are identified and kernel density estimation is applied to estimate the probability density functions associated with ChIP and control read locations. For both ChIP and control reads, we estimate probability densities functions for forward reads (reads that align to the forward strand) and reverse reads (reads that align to the reverse strand). To identify enriched regions, we divide the genome into nonoverlapping bins into which reads are binned, and we search for significantly enriched bins by using a log-likelihood ratio (LLR) test. The probability density functions associated with ChIP and control read locations are used to derive enrichment profiles that describe the enrichment level throughout each enriched region for both forward and reverse reads.

From the enrichment profiles, an initial estimate is made of the shape of an enrichment peak. Specifically, we use a heuristic that searches for narrow peaks of large amplitude. This peak shape is used to deconvolve the enrichment profiles (that is, binding site locations and magnitudes are estimated under the assumption that each binding site gives rise to one peak of the given shape). Because the initial peak-shape estimate may be incorrect, the binding-site locations and magnitudes thus obtained are used to reestimate and refine the peak shape. We then return to estimating binding-site locations and magnitudes by using the reestimated peak shape. We repeat this iterative cycle until the change in the peak shape achieved in an iteration is negligible.

### Performance

To test CSDeconv, we applied it to novel ChIP-seq data for the DosR transcription factor in MTB and to existing data for the GABP transcription factor in humans.

DosR is a transcription factor that is believed to play an important role in MTB virulence, and it is therefore important to understand its targets and mechanism of operation. The dosR locus is among the first induced by reduced oxygen [20-22] or low levels of nitric oxide [23], which are conditions thought to reflect *in vivo *infection. Moreover, DosR is induced rapidly on infection of macrophages [24,25] and mice [23,26]. DosR is therefore believed to play an important role in infection, and it is necessary for hypoxic gene induction [16] - a condition used to promote nonreplicating persistence *in vitro*. Thus, DosR has received significant attention, and a putative motif has been derived for its binding site [16].

GABP is a human transcription factor that was previously studied by using ChIP-seq by Valouev and colleagues [4]. The potential for GABP to bind multiple times in closely spaced regions [27] makes it a suitable test case for blind deconvolution to tease apart multiple binding sites over short distances. As it is currently implemented, CSDeconv cannot be used straightforwardly to analyze genome-wide binding of GABP because the computational requirements of CSDeconv prohibit the analysis of such a large number of enriched regions. CSDeconv can, however, be applied to analyze a subset of all enriched regions, thus demonstrating the efficacy of blind deconvolution, even in the lower sequencing depths that are achieved on mammalian genomes.

To apply CSDeconv effectively, it is necessary to set its parameters to achieve an appropriate level of sensitivity and specificity. Two parameters of principal importance exist: the threshold on the LLR that is used to determine significantly enriched bins, and the regularization factor *α *that determines the number of binding sites that are called in an enriched region. We determine appropriate levels for these parameters by estimating the false discovery rate (FDR) achieved by various settings. The FDR is estimated by using the same procedure used in a number of ChIP-seq and ChIP-chip peak finders [7,28,29]: a sample swap. ChIP and control reads are swapped, CSDeconv is run, and the empirical FDR is calculated as the number of detections in the control (over ChIP) sample divided by the number of detections in the ChIP (over control) sample.

**Figure 2:**
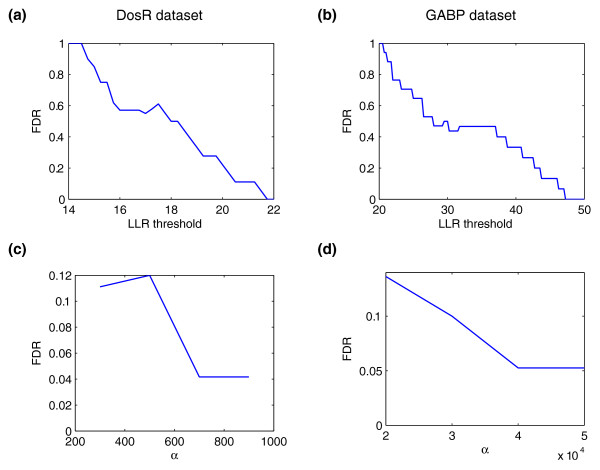
**Empirical FDR of CSDeconv**. **(a, b) **The empirical FDR for enriched regions as a function of LLR threshold is shown for the **(a) **DosR and **(b) **GABP datasets. **(c, d) **With the LLR threshold fixed, the empirical FDR for binding sites as a function of the regularization factor α is shown for the **(c) **DosR dataset (LLR threshold at 18.75) and the **(d) **GABP dataset (LLR threshold at 38.5).

In Figures 2a and 2b, we show the empirical FDR for enriched regions as a function of the LLR threshold for the DosR and GABP datasets, respectively. We see that, owing to its lower coverage, larger LLR thresholds are required to achieve low FDRs in the GABP dataset. To ensure that a sufficient number of false enriched regions exist to obtain a good estimate of the FDR for binding sites, we set the LLR threshold to achieve a relatively high empirical FDR for enriched regions. We set the LLR threshold to 18.75 for the DosR dataset and 38.5 for the GABP dataset, achieving empirical FDRs for enriched regions of 0.389 and 0.40, respectively.

At these LLR thresholds, we then determine the empirical FDR for binding sites at various levels of *α*. The empirical FDR for binding sites as a function of *α *is shown in Figures 2c and 2d. For the results we report, we set *α *to 700 for the DosR dataset and to 40,000 for the GABP dataset, achieving low empirical FDRs of 0.042 and 0.044, respectively.

**Figure 3:**
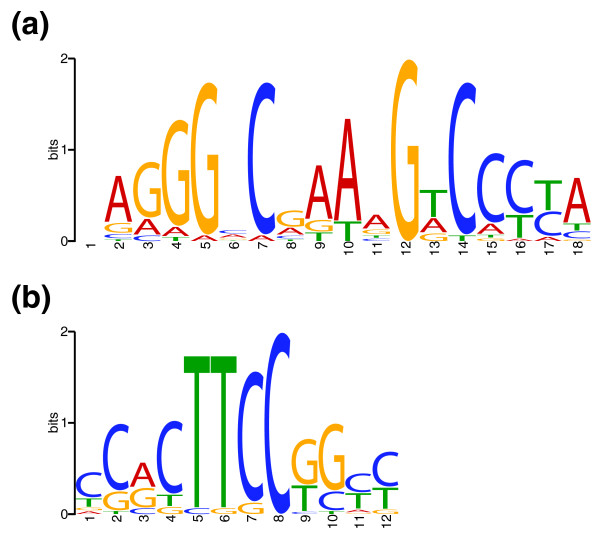
**Sequence logos of binding motifs**. The sequence logo of the binding motifs found through CSDeconv analysis is shown for **(a) **DosR and **(b) **GABP.

For DosR, CSDeconv identified a total of 24 binding locations (see Table 1). With MEME [30], we searched for a conserved DNA motif within 50 bp of the binding locations, and we found an 18-bp motif that closely matches the motif previously identified by Park and co-workers [16] from expression analysis (see Figure 3a). Then, by using MAST [31], we searched for the presence of this motif within 50 bp of the binding locations and, for 23 of the 24 binding locations, we found a matching sequence. The average difference of the position estimated by CSDeconv and the center of the motif-matching sequence is 13.9 bp, and the average absolute difference is 20.1 bp. An examination of the sequences in the 18 enriched regions in which these 24 binding sites occurred did not reveal any likely binding sites that were not called.

**Table 1:** Results of CSDeconv on DosR data

Peak ID	Position	Amplitude	Position of motif match	Difference	Absolute difference	Location
1	88,097.4	2.4	88,125.5	28.1	28.1	Upstream of Rv0079
2	434,899.0	2.8	434,926.5	27.5	27.5	In Rv0356c; upstream of Rv0355c
3	665,844.1	7.8	665,861.5	17.4	17.4	In Rv0573c; upstream of Rv0572c
4	668,490.6	2.8	668,497.5	6.9	6.9	Upstream of Rv0574c
5	801,443.4	3.6	801,480.5	37.1	37.1	In Rv0702
6	1,639,602.3	8.5	1,639,627.5	25.2	25.2	In Rv1453
7	1,960,515.3	13.7	1,960,520.5	5.2	5.2	Upstream of Rv1733c
8	1,960,611.5	28.0	1,960,624.5	13	13	Upstream of Rv1733c
9	1,960,697.2	11.0				Upstream of Rv1733c
10	1,965,459.6	10.8	1,965,471.5	11.9	11.9	Upstream of Rv1737c
11	1,965,540.8	15.2	1,965,511.5	-29.3	29.3	Upstream of Rv1737c
12	2,056,358.2	2.7	2,056,375.5	17.3	17.3	Upstream of Rv1813c, Rv1814
13	2,238,941.0	9.5	2,238,938.5	-2.5	2.5	Upstream of Rv1996
14	2,256,459.3	13.0	2,256,496.5	37.2	37.2	Upstream of Rv2007c
15	2,278,994.7	43.5	2,279,005.5	10.8	10.8	Upstream of Rv2031c, Rv2032
16	2,279,049.0	25.8	2,279,062.5	13.5	13.5	Upstream of Rv2031c, Rv2032
17	2,949,475.3	5.7	2,949,472.5	-2.8	2.8	Upstream of Rv2623
18	2,953,044.8	8.3	2,953,074.5	29.7	29.7	Upstream of Rv2626c
19	2,954,750.4	5.1	2,954,792.5	42.1	42.1	Upstream of Rv2627c, Rv2628
20	2,955,065.5	9.9	2,955,031.5	-34	34	Upstream of Rv2627c, Rv2628
21	2,955,479.2	9.5	2,955,476.5	-2.7	2.7	Upstream of Rv2629
22	3,492,069.6	12.0	3,492,092.5	22.9	22.9	In Rv3126c; upstream of Rv3127
23	3,496,438.8	54.7	3,496,451.5	12.7	12.7	Upstream of Rv3130c, Rv3131
24	3,500,822.1	3.6	3,500,853.5	31.4	31.4	Upstream of Rv3134c

CSDeconv identifies a total of 24 binding sites. The position of the sequence matching the motif shown in Figure 3a is given if such a sequence exists within 50 bp of the predicted binding site.

**Figure 4:**
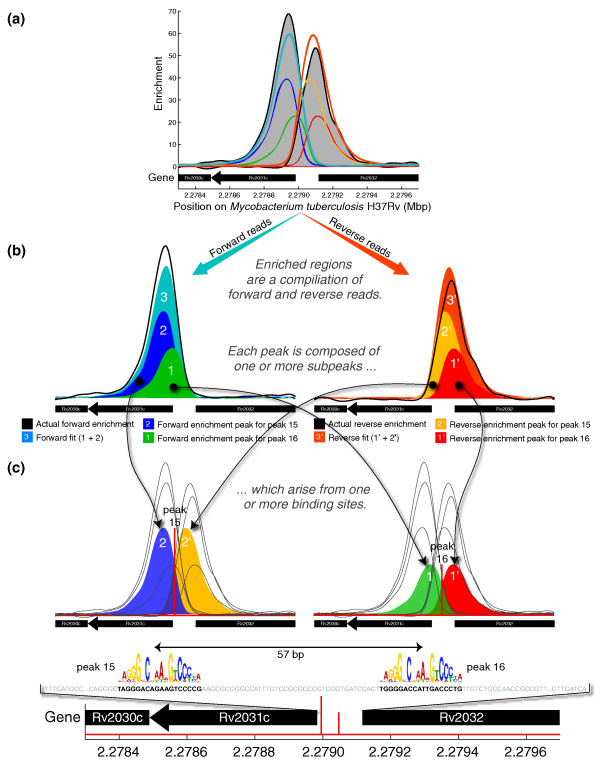
**Illustration of the results obtained by CSDeconv for DosR binding upstream of Rv2031c**. **(a) **The forward and reverse enrichment profiles obtained after kernel density estimation of the read distributions are shown in black and shaded in gray. Colored lines display various fits arising from estimated binding. Note that no distinct peaks are evident in the enrichment profiles and, in particular, there are no dips. **(b) **Both forward and reverse reads are associated with fits: the forward fit 3 is the sum of the forward enrichment peaks 1 and 2, whereas the reverse fit 3' is the sum of the reverse enrichment peaks 1' and 2'. **(c) **The combined forward and reverse enrichment peaks arise from two binding sites, which are peaks 15 and 16 in Table 1. Motif logos overlay the actual sequence of the intergenic region truncated for brevity, showing the two binding sites, which are separated by a scant 57 bp. Enrichment is plotted as the fold magnitude of the ChIP read density over the control read density.

Notably, we are able to identify several instances of very closely spaced binding sites. For example, we identify two binding sites upstream of Rv1737c that are separated by only 40 bp, and we identify two binding sites upstream of Rv2031c that are separated by only 57 bp. As an illustrative example, we show the latter in Figure 4. That binding occurs at both of these sites was previously established by mobility-shift assays [16]. Moreover, our algorithm predicts that more binding occurs at the more upstream of the two sites, which is the site that has been found to be responsible for a greater fraction of the DosR-dependent induction of Rv2031c under hypoxic conditions.

For GABP, we applied CSDeconv to an arbitrarily chosen 2-Mbp segment of human chromosome 19 that starts from chromosome position 60,000,000. In this segment, we identified 23 GABP-binding locations (see Table 2) of which 17 (74%) lie within CpG islands, indicative of promoter and control regions [32]. With the same analysis as for DosR, we found a 12-bp motif resembling that previously identified [4,19] that lies within 50 bp of 18 of the 23 binding locations found by CSDeconv (see Figure 3b). The average difference of the position estimated by CSDeconv and the center of the motif-matching sequence is 9.1 bp, and the average absolute difference is 23.5 bp. Again, we identify several instances of very closely spaced binding sites. In particular, we identify two binding sites located at positions 60,209,299.5 and 60,209,319.5 that are separated by a mere 20 bp.

**Table 2:** Results of CSDeconv on GABP data

Peak ID	Position	Amplitude	Position of motif match	Difference	Absolute difference	Location	CpG island
1	60,209,266.5	145.6	60,209,299.5	33	33	Upstream of Hs.631589	No
2	60,209,352.0	242.4	60,209,319.5	-32.5	32.5	Upstream of Hs.631589	No
3	60,209,441.8	32.1	60,209,417.5	-24.3	24.3	In Hs.631589	No
4	60,355,290.1	93.1	60,355,272.5	-17.6	17.6	In TNNI3	No
5	60,355,384.7	109.8	60,355,373.5	-11.2	11.2	In TNNI3	No
6	60,483,301.5	116.7	60,483,320.5	19	19	Upstream of HSPBP1	Yes
7	60,508,823.3	53.5	60,508,860.5	37.2	37.2	In BRSK1	No
8	60,589,079.4	89.0	60,589,061.5	-17.9	17.9	In LOC388564, Upstream of RPL28	Yes
9	60,784,129.0	152.4	60,784,167.5	38.5	38.5	Upstream of ZNF579	Yes
10	60,802,703.2	72.5	60,802,723.5	20.3	20.3	In FIZ1	Yes
11	60,802,828.5	34.2				Upstream of FIZ1	Yes
12	60,808,717.3	19.1	60,808,762.5	45.2	45.2	Downstream of ZNF524	Yes
13	60,838,116.9	163.1				Upstream of ZNF580	Yes
14	60,838,170.7	87.4	60,838,194.5	23.8	23.8	Upstream of ZNF580	Yes
15	60,846,738.1	37.1				Upstream of ZNF581	Yes
16	60,856,978.7	23.4				Upstream of U2AF2	Yes
17	60,878,268.6	125.4	60,878,250.5	-18.1	18.1	Upstream EPN1	Yes
18	60,878,368.7	105.4	60,878,386.5	17.8	17.8	Upstream of EPN1	Yes
19	61,517,917.1	24.8				Upstream of LOC729994	Yes
20	61,518,034.1	98.8	61,518,049.5	15.4	15.4	Upstream of LOC729994	Yes
21	61,518,366.6	26.5	61,518,358.5	-8.1	8.1	Upstream of LOC729994	Yes
22	61,741,681.4	16.8	61,741,681.5	0.1	0.1	In ZFP28	Yes
23	61,741,850.8	25.5	61,741,894.5	43.7	43.7	In ZFP28	Yes

CSDeconv identifies a total of 23 binding sites between position 60,000,000 and 62,000,000 on chromosome 19. The position of the sequence matching the motif shown in Figure 3a is given if such a sequence exists within 50 bp of the predicted binding site.

### Comparison with other methods

Other methods for ChIP-seq data analysis search for peaks of enrichment and call such peaks as single binding sites. They do not deconvolve the peaks into separate binding sites. As such, they are generally incapable of identifying closely spaced binding sites where enrichment peaks overlap and merge into a single peak, as is the case, for example, in Figure 4. We therefore expect that, for the same number of binding sites called, CSDeconv will exhibit a greater level of accuracy than alternative methods, which are based on peak searching. Such alternative methods will miss instances of closely spaced binding sites and instead call false binding sites.

We demonstrate the capabilities of CSDeconv by comparing it with MACS [7] and SISSRs [9], two publicly available ChIP-seq peak-finding methods. For both DosR and GABP, we use MEME and MAST to determine the percentage of predicted binding sites that have an associated motif within 50 bp for CSDeconv, MACS, and SISSRs, applied at varying levels of stringency.

**Figure 5:**
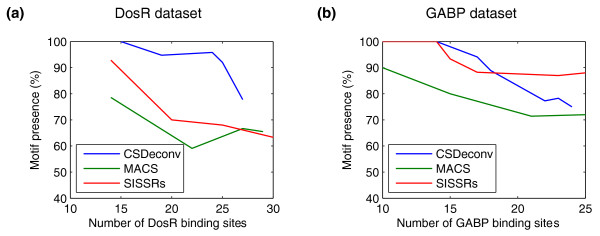
**Comparison of CSDeconv, MACS, and SISSRs by motif analysis**. The percentage of predicted binding sites with associated motifs within 50 bp is shown as a function of the number of predicted binding sites with CSDeconv, MACS, and SISSRs for **(a) **DosR and **(b) **GABP. For MACS and SISSRs, we take the predicted binding-site location to be the peak center.

For the DosR dataset, CSDeconv consistently yields a significantly higher percentage of motif occurrences than do both MACS and SISSRs (see Figure 5). The results we show are obtained with the LLR threshold fixed at 18.75, as before, and we vary *α *to obtain differing numbers of predicted binding sites. Thus, we expect the accuracy to fall off rapidly after a certain number of predicted sites are called, because the number of enriched regions remains constant. The decline in accuracy is observably noticeable after approximately 25 sites. For the GABP dataset, CSDeconv yields a higher percentage of motif occurrences than MACS and is comparable in performance to SISSRs. The LLR threshold is fixed at 38.5, as before, so we again expect the accuracy to fall off rapidly for CSDeconv.

Motif occurrence can be used not only to validate binding sites, but potentially also to find them. It may be possible to avoid blind deconvolution by simply searching for multiple, rather than single-motif occurrences around a ChIP-seq peak. To establish that the performance improvements observed in CSDeconv are due to blind deconvolution and cannot simply be found by motif searching, we compared CSDeconv against a "simplified" version; instead of using blind deconvolution to detect instances of multiple binding sites at a single enriched region, we simply used MEME to search for conserved motifs that can occur arbitrarily many times around peaks in each enriched region. The results of this analysis are shown in Table 3. We see that there are both instances in which binding sites are called by CSDeconv and not be the simplified version and *vice versa*. In general, the simplified CSDeconv calls more binding sites, and this is especially true in the case of GABP, where the motif is less informative. Cases exist, however, in which the simplified CSDeconv fails to call binding sites that are called by CSDeconv. These cases are supported by read enrichment, and slight modifications to the motif are usually enough to allow a match at those locations, but the simplified CSDeconv has difficulty finding a suitable motif. As for whether the additional binding sites called by the simplified CSDeconv are false positives, this is difficult to determine, as few true negatives are known, especially when it comes to closely spaced binding sites. In the case of the *acr *(Rv2031c) gene in MTB, however, the binding site in this gene's promoter region that is called by the simplified CSDeconv and is not called by CSDeconv (at position 2279027) is unlikely to be bound by DosR at any significant level, based on previous studies [16]. We conclude, therefore, that the results obtained by CSDeconv cannot simply be obtained by motif searching, and our results indicate that the latter method results in a higher rate of false positives.

**Table 3:** Comparison of CSDeconv with "simplified" CSDeconv

**DosR data**		**GABP data**	
	
**Motif match for CSDeconv**	**Motif match for simplified CSDeconv**	**Motif match for CSDeconv**	**Motif match for simplified CSDeconv**
	
88,125.5	88,124.5		60,209,242
434,926.5	434,925.5		60,209,279
665,861.5	665,860.5	60,209,300	60,209,299
	665,882.5	60,209,320	60,209,319
668,497.5	668,498.5		60,209,356
801,480.5			60,209,397
1,639,628	1,639,629	60,209,418	60,209,417
1,960,521	1,960,520	60,355,273	60,355,274
	1,960,542		60,355,294
1,960,625	1,960,624		60,355,314
1,965,472	1,965,473		60,355,334
1,965,512	1,965,513	60,355,374	60,355,375
	1,965,533		60,355,395
2,056,376	2,056,377		60,355,415
	2,056,410		60,355,435
	2,238,919	60,483,321	60,483,320
2,238,939	2,238,940		60,508,762
	2,256,475	60,508,861	60,508,862
2,256,497	2,256,498	60,589,062	60,589,061
2,279,006	2,279,007		60,589,119
	2,279,027		60,589,144
2,279,063	2,279,062		60,589,163
2,949,473	2,949,472		60,784,040
	2,949,496		60,784,108
2,953,075	2,953,074	60,784,168	60,784,167
	2,953,099		60,784,201
2,954,793			60,802,658
2,955,032	2,955,033		60,802,702
	2,955,099	60,802,724	60,802,725
2,955,477			60,802,741
3,492,093	3,492,094		60,802,761
3,496,452	3,496,453		60,802,785
	3,500,832		60,802,817
3,500,854	3,500,853		60,808,690
		
			60,808,717
		60,808,763	
			60,808,770
			60,808,789
			60,808,815
		60,838,195	60,838,196
			60,846,664
			60,846,794
			60,857,070
		60,878,251	60,878,250
			60,878,292
			60,878,366
		60,878,387	60,878,388
			61,517,979
		61,518,050	61,518,051
		61,518,359	
		61,741,682	
			61,741,862
		61,741,895	
			61,741,918

Locations of motif matches for CSDeconv and a simplified CSDeconv that uses motif finding instead of blind deconvolution are listed for both the DosR and GABP datasets. Offsets are observed in the matching sequences for the two methods owing to differing motifs found by the methods.

## Conclusions

As sequencing becomes faster and cheaper, ChIP-seq will likely become the method of choice for mapping sites of protein-DNA interaction, and methods that can call such sites effectively and accurately from ChIP-seq data will become increasingly important. CSDeconv allows accurate calls to be made in the case of closely spaced transcription factor-binding sites, which is a phenomenon observed frequently, particularly in prokaryotes. The method we use differs substantially from previous techniques in that we use a blind-deconvolution approach, explicitly estimating the shape of an enrichment peak in addition to binding-site locations and magnitudes, thereby distinguishing closely spaced transcription factor-binding sites.

As it is currently implemented, CSDeconv is not attractive for the study of genome-wide binding of transcription factors in mammalian genomes because of its computational requirements. We have, however, demonstrated that CSDeconv can be applied to mammalian ChIP-seq data and is useful for the analysis of such data. Although it is difficult to predict how the number of iterations required by CSDeconv will increase as the number of enriched regions increases, each iteration simply scales linearly. Thus, whereas CSDeconv is currently suited to handle a small number (tens) of enriched regions, it is likely that, with algorithmic improvements, blind deconvolution can be straightforwardly applied to study genome-wide protein-DNA interaction in mammals and other eukaryotes.

## Abbreviations

bp: base pair(s); ChIP: chromatin immunoprecipitation; ChIP-Seq: chromatin immunoprecipitation coupled with massively parallel sequencing; DosR: dormancy survival regulator; FDR: false discovery rate; FWHM: full width at half maximum; GABP: growth-associated binding protein; LLR: log-likelihood ratio; MTB: *Mycobacterium tuberculosis*.

## Authors' contributions

DSL and JEG conceived the project. DSL designed and implemented the algorithm. DSL and BW performed the experiments and wrote the article. AS and DRS generated the DosR dataset.
